# Epigenetic Modifications in Stress Response Genes Associated With Childhood Trauma

**DOI:** 10.3389/fpsyt.2019.00808

**Published:** 2019-11-08

**Authors:** Shui Jiang, Lynne Postovit, Annamaria Cattaneo, Elisabeth B. Binder, Katherine J. Aitchison

**Affiliations:** ^1^Department of Medical Genetics, University of Alberta, Edmonton, AB, Canada; ^2^Department of Oncology, University of Alberta, Edmonton, AB, Canada; ^3^Biological Psychiatric Unit, IRCCS Istituto Centro San Giovanni di Dio Fatebenefratelli, Brescia, Italy; ^4^Department of Translational Research in Psychiatry, Max Planck Institute of Psychiatry, Munich, Germany; ^5^Department of Psychiatry and Behavioral Sciences, Emory University School of Medicine, Atlanta, GA, United States; ^6^Department of Psychiatry, University of Alberta, Edmonton, AB, Canada

**Keywords:** childhood trauma, stress disorders, mental health, the hypothalamic-pituitary-adrenal axis (HPA), epigenetic association studies

## Abstract

Adverse childhood experiences (ACEs) may be referred to by other terms (e.g., early life adversity or stress and childhood trauma) and have a lifelong impact on mental and physical health. For example, childhood trauma has been associated with posttraumatic stress disorder (PTSD), anxiety, depression, bipolar disorder, diabetes, and cardiovascular disease. The heritability of ACE-related phenotypes such as PTSD, depression, and resilience is low to moderate, and, moreover, is very variable for a given phenotype, which implies that gene by environment interactions (such as through epigenetic modifications) may be involved in the onset of these phenotypes. Currently, there is increasing interest in the investigation of epigenetic contributions to ACE-induced differential health outcomes. Although there are a number of studies in this field, there are still research gaps. In this review, the basic concepts of epigenetic modifications (such as methylation) and the function of the hypothalamic-pituitary-adrenal (HPA) axis in the stress response are outlined. Examples of specific genes undergoing methylation in association with ACE-induced differential health outcomes are provided. Limitations in this field, e.g., uncertain clinical diagnosis, conceptual inconsistencies, and technical drawbacks, are reviewed, with suggestions for advances using new technologies and novel research directions. We thereby provide a platform on which the field of ACE-induced phenotypes in mental health may build.

## Adverse Childhood Experiences/Childhood Trauma

Stressful or traumatic events experienced in childhood or adolescence can be driven by a broad range of life events, including but not limited to physical injury, natural disaster, bullying, and childhood maltreatment (1). They are referred to by many terms, including early life adversity, early life stress, early life trauma, and adverse childhood experiences (ACEs) (2). It is reported that the worldwide average trauma exposure rate is 69.7% for children and adults (3). In the United States, around 60% of adults reported that they had experienced at least one type of ACE (2).

ACEs/childhood trauma are associated with negative health outcomes, both mentally and physically (4). Individuals exposed to multiple types of childhood trauma show an increased risk of early mortality, which decreases their lifespan up to 20 years (5). Physically, childhood trauma has been associated with increased risk of cardiovascular disease (6), autoimmune disease (7), gastrointestinal symptoms (8), poor dental health (9), obesity, and type 2 diabetes (10). Psychologically, childhood trauma is regarded as one of the major risk factors for psychopathology. Childhood trauma has been associated with many mental disorders (11). Specifically, childhood trauma has been linked to posttraumatic stress disorder (PTSD) (12), insomnia (13), anxiety (14), depression (15, 16), bipolar disorder (17, 18), maladaptive daydreaming (MD) (19), hallucinations (20), borderline personality disorder (21), disruptive behavior (22), risky behaviors (23, 24), substance abuse (25, 26), antisocial behavior (27), and eating disorders (28, 29).

Childhood trauma impacts children to different extents. Some people are more vulnerable, whereas, others show the characteristic of “resilience,” with the ability to “bounce back” even after adversity (30). Multiple factors, e.g., genetic, epigenetic, and environmental factors, and their interactions contribute to the differential health outcomes induced by childhood trauma. According to a neural diathesis-stress model, genetic predisposition and environmental factors contribute synergistically to the development of mental disorders. The magnitude of the heritability of a phenotype is one way of estimating the relative magnitude of the genetic contribution. In the case of ACE-associated psychiatric disorders such as PTSD, the heritability is in fact low to moderate (31). Similarly, the heritability of resilience is low to moderate, varying in research reports from 25% to 60% (32–34). These heritability values suggest that there may be other mechanisms contributing to these phenotypes, such as gene by gene interaction and gene by environment interactions, and epigenetic mechanisms. Consequently, it might well be productive to explore genetic, epigenetic, and environmental interactions in resilience and ACE-associated health outcomes.

## The Associations Between Genetic and Epigenetic and Childhood Trauma

### Epigenetic Modifications

The human genome is made up of DNA, which stands for deoxyribonucleic acid, the genetic code which is a continuous sequence of four “letters” or “bases,” A, G, C, T (A = adenine, C = cytosine, G = guanine, T = thymine). This encodes heritable information from parents to offspring. Part of this sequence is “read” during a process known as “transcription.” Transcriptional machines, which have a complicated structure and are made up of several protein subunits, are needed to start this process. Following transcription of the locus, the noncoding DNA areas (known as “introns”) are spliced out and the regions that are coding proteins/peptides, known as “exons,” are converted into mRNA sequences. These mRNAs are then used to build different protein structures from “building blocks” known as amino acids. In the next, “posttranslational,” stage, further modifications may occur and influence the function of the protein. In general, gene expression is a complicated dynamic process and controlled by diverse regulators at different levels, such as transcriptional regulation (*cis*: e.g., promoters, *trans*: e.g., DNA-binding proteins), RNA processing (RNA splicing, noncoding RNA, miRNAs, etc.), translational regulation, and posttranslational regulation (acetylation, phosphorylation, and glycation, etc.) (35).

Epigenetic modifications regulate this dynamic process of DNA to protein. Epigenetics, which means “outside conventional genetics” (36), focuses on the regulation of “turning on or off” genes without changing the DNA sequence, but rather the accessibility of regulatory transcription factors to the gene. Epigenetic modifications impact on multiple nuclear processes, such as DNA packaging and chromatin structure, and thus on gene expression, with various directions of effect (which may be conceptualized as “epigenetic readers, writers, and erasers”) (37). Such modifications include changes in the spatial positioning of chromosomal territories (38). There are three main types of epigenetic modifications: DNA methylation, histone modifications, and various RNA-mediated processes (39, 40). Epigenetic modifications may be cell-type-specific.

Cytosine methylation (5-methylcytosine, 5-mC) is very common in both eukaryotes and prokaryotes (41). It largely happens at cytosine followed by guanine residues (CpG) sites; less commonly, cytosine may be methylated at CpA, CpT, or CpC sites. A family of enzymes named DNA methyltransferases (DNMTs) regulates DNA methylation through transferring a methyl group to the DNA base cytosine (42). Methylation, which is similar to a protective cover on the DNA, generally suppresses gene expression by physically preventing transcription factor binding (43). It also suppresses gene expression by interacting with other mechanisms, e.g., histone deacetylase (HDACs) complex recruitment. For example, methyl-CpG-binding proteins (MeCP) 2 binds tightly to chromatin in a methylation-dependent way, which induces the formation of the histone deacetylase complex. This complex induces transcriptional suppression by changing chromatin structures (44). However, DNA methylation also enhances gene expression through more complicated mechanisms such as the methylation of CCCTC-binding factor (CTCF) binding sites and/or intronic regions (45–49). Hydroxymethylcytosine (5-hmC) is another form of DNA methylation. It is in fact converted from5-mC through an oxidative reaction, by the ten-eleven translocation methylcytosine dioxygenase (TET) 1 enzyme. Similarly, 5-hmC is able to both activate and suppress gene expression in a bidirectional manner (50). The expression of 5-hmC is highly concentrated in the brain and is dynamic during fetal development (51). It has been reported to play important roles in neuronal function, learning and memory, and stress-mediated responses (52).

As for histone modification, it impacts chromatin structure and DNA packaging (37) [e.g., the acetylation of histones may render DNA more or less accessible to transcription factors, leading to enhanced or reduced transcriptional activity (53)]. It has been estimated that the full length of DNA is more than 500 billion kilometres in the human body (54). Consequently, DNA needs to be packed tightly into cells. Histones are directly involved in the packaging process. Basically, a core of DNA (around 146 base pairs) wraps around each histone to form a structure known as a “nucleosome.” Subsequently, an octamer comprising four different histones (H3, H4, H2A, and H2B) is formed. This is further packed into chromatin fibres and then into chromosomes, a unit now visible under a light microscopes. There are several types of histone modifications, including acetylation, methylation, phosphorylation and ubiquitylation. The specific modification patterns of histones, which are called histone codes, work with the other chromatin associated proteins, change the structure of the local chromatin, and thus impact the process of gene expression, such as transcription, replication and DNA repair. The proper topological structure of chromatin is essential in gene expression and genome maintenance (55).

Lastly, noncoding small RNAs (e.g., miRNAs) are also able to mediate sequence-specific modulation of gene expression by different mechanisms (56). For example, miRNAs bind to their target mRNAs *via* complementary sequences, which induces the cleavage and degradation of the corresponding mRNA (57). More recently, additional epigenetic modifications have been discovered, including for example, RNA methylation (58).

Each cell in the living organism, under normal conditions, essentially shares the same copy of DNA, but eventually develops and differentiates to different cell types under regulatory mechanisms. Epigenetic modifications such as genetic imprinting (59) are necessary for embryogenesis and gametogenesis (60, 61), differentiation, and development. In fact, epigenetic regulation occurs throughout the lifespan and can be induced by random changes (62) or by multiple different environmental factors (63). For example, changes in human epigenome have been associated with processes related to adaptation and evolution (64, 65), and have also been linked to several diseases, such as cancer (66), type 2 diabetes (67), and autoimmune rheumatic disorders, such as systemic lupus erythematosus (SLE) (68). Epigenomic alterations are also associated with pathologies characterized by behavioral or/and cognitive problems, e.g., Alzheimer’s disease (69), Rett syndrome (70), Cushing’s syndrome (71), depression (72), addiction (73), aggression and antisocial behavior (74), and also with illnesses characterized by childhood trauma exposures, such as mental disorders (75).

Early life is a special period characterized by a high level of plasticity and fast development (76). Thus, the impact of childhood trauma is particularly deleterious, since the developmental trajectory of the brain is affected, with resultant alteration of the circuitry for threat detection, emotional regulation, and the reward system (77).

In this paper, we will focus on the epigenetic modification of DNA methylation, as this has the most data relevant to childhood trauma.

### The Associations Between Methylation and Childhood Trauma

#### Stress and the HPA Axis

Why does childhood trauma impact health outcomes? One mechanism is by the induction of toxic stress. In fact, stress can be classified into “good stress,” “bearable stress,” and “toxic stress” (78), and has acute, delayed and long-term effects on the body (79). “Good stress” can be coped with by physiological mechanisms, encouraging healthy growth; “bearable stress” states may eventually be turned into homeostasis through successful interventions; whereas, “toxic stress,” which is characterized by prolonged or frequent activation and dysregulation of the stress response pathway, induces long-term changes and damage not only to the brain but also to the rest of body (2, 80).

**Figure 1:**
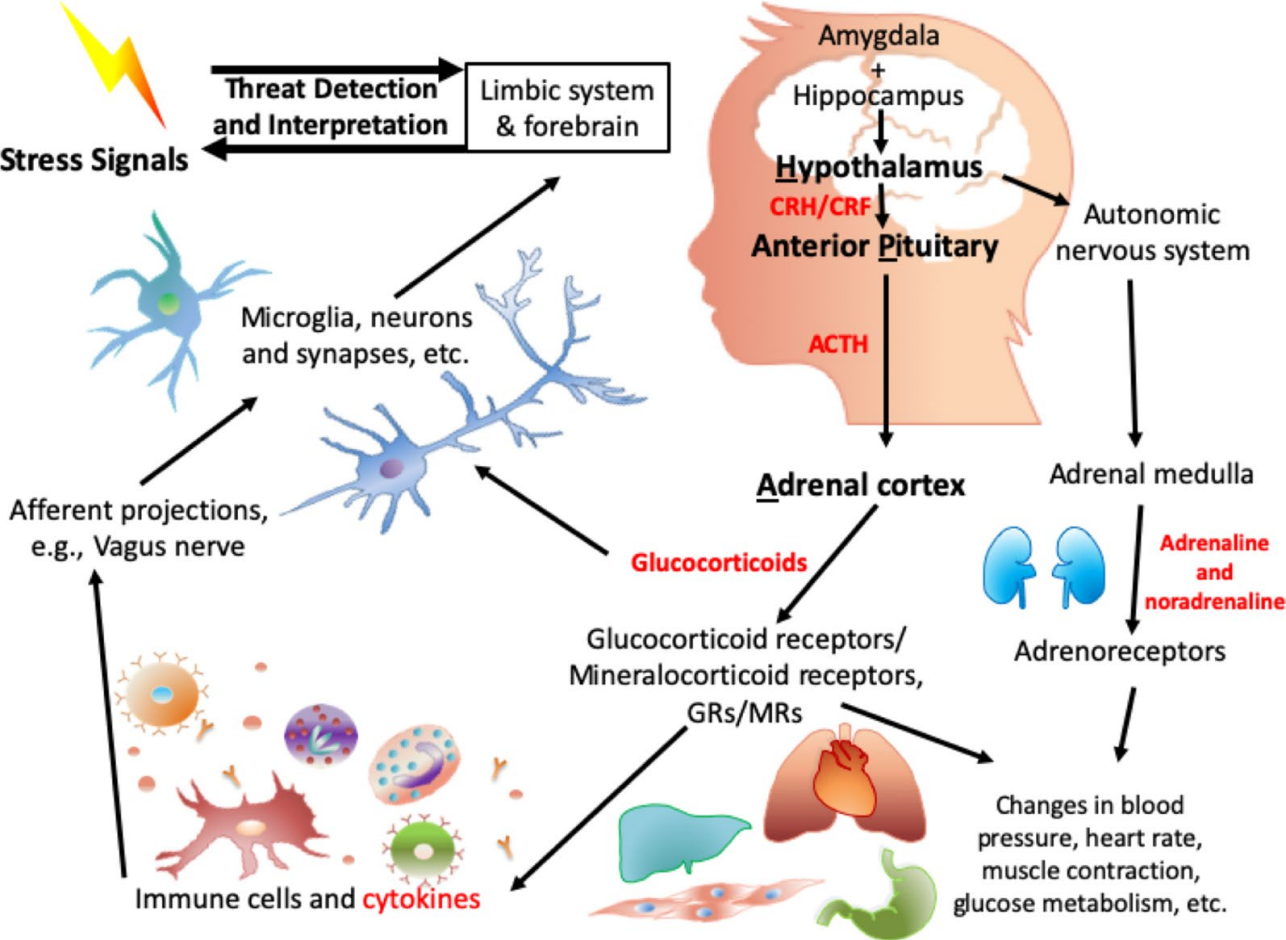
Biological mechanisms involved in HPA axis induced dysregulation in response to toxic stress.

The central biological pathway involved in the response to stress is the hypothalamic-pituitary-adrenal (HPA) axis (**Figure 1**). In 1914, Walter B. Cannon put forward the “fight or flight” model, which described the body’s response toward stress (81). Around the 1950s, Selye’s general adaptation syndrome was put forward: that chronic stress could induce a nonspecific response in the body, such as increased heart rate and blood pressure (82). More recently, more in-depth research has illustrated that alterations in the HPA axis have been associated with the process of dealing with stress, especially toxic stress-induced negative health outcomes (83).

When a threat signal is recognized, the central nervous system (CNS): amygdala (84), hypothalamus (85), and parts of brainstem such as the locus coeruleus, (86–88), which are regarded as the central components of the stress response, will be activated. Neurotransmitters such as glutamate, serotonin (89), and γ-aminobutyric acid (GABA) are involved in this signal transmission. On receipt of the neuronal signal from the amygdala and locus coeruleus, numerous neuropeptides are released from the hypothalamus, including arginine vasopressin (AVP) and stress-induced corticotropin-releasing factor/hormone (CRF/H) (90). The CRF Receptor 1 (CRFR_1_) on the anterior pituitary is activated, which results in the secretion of adrenocorticotropic hormone (ACTH). AVP works together with CRH to contribute to the ACTH response (91). ACTH acts on receptors in the adrenal cortex, leading to the release of stress-related hormones: glucocorticoids (cortisol) and mineralocorticoids (aldosterone). These stress-related hormones mediate the stress response (92) to induce changes in heart rate, blood pressure, metabolism (93), and immune function (94). Other neuropeptides/neurotrophic factors, such as neuropeptide Y (95), dynorphin (96), and oxytocin as well as brain-derived neurotrophic factor (BDNF), are also involved in the HPA axis and in the orchestration of the response to stress.

On the other hand, in the sympathetic adrenal medullary (SAM) axis, a signal from the hypothalamus activates the adrenal medulla, and then induces the secretion of the catecholamines adrenaline and noradrenaline. Peripheral organs (e.g., heart, liver), glands, and vessels have receptors for these hormones and are in addition regulated by the sympathetic autonomic neurons. Together with the HPA axis as mentioned above, the downstream effects, e.g., increased heart rate and blood pressure, are intended to be biologically adaptive, to enhance the individual’s ability to respond to the stressor.

Importantly, cortisol provides negative feedback are the level of the hypothalamus (97) to stop the HPA axis from being excessively activated with consequent deleterious health effects. In addition, within the autonomic nervous system, parasympathetic neurons balance the activation of the sympathetic system, inducing a “rest and digest” body state. Childhood stress and trauma alter the HPA axis (98) and the long-term dysregulation of the HPA axis induced by childhood stress/trauma has been associated with increased risk of adverse health outcomes. For some of these, the effects of adversity appears to be dose-dependent (99–101).

#### Hotspot Genes

There is increasing interest in the investigation of epigenetic and environmental interactions in ACE-induced differential health outcomes. In humans, studies have mainly focused on peripheral tissues, such as peripheral blood, buccal cells, or saliva. There are also studies with human postmortem brain tissue. For example, Labonte and colleagues reported that in hippocampal tissues derived from those who had died by suicide, comparing those with and without a history of childhood abuse, there were 362 differentially methylated promoter sites. Among these, 248 sites were hypermethylated and 114 were hypomethylated (102). Similarly, there was a bidirectional regulation of methylation in the cingulate cortex of those with/without childhood trauma who has had depression and died by suicide, with the highest differential methylation occurring in genes that related to myelin (103). In a 2017 systematic review of epigenetic associations with childhood trauma in first episode psychosis patients and healthy individuals, childhood trauma was associated with global hypomethylation in peripheral blood samples (104, 105).

A key limitation of such epigenetic research as described above is nonetheless the tissue specificity of effects, which means that for only very limited sites scan congruent changes across tissues be expected (106, 107). In fact, even with the same sample, e.g., saliva taken at different times from the same individual, the cellular composition (proportion of different cells) may vary, which brings challenges to the analysis of methylation results (108).

Relevant biological systems relevant to the HPA axis are summarized in **Figure 1** with highlights provided below.

##### FKBP5

The *FKBP5* gene encodes a heat shock protein 90 (HSP90) cochaperone that modifies the sensitivity of steroid receptor hormones, interacting with the glucocorticoid receptor (GR), the progesterone receptor (PR), and the androgen receptor (AR). Together with other chaperone proteins such as Hsp90, FKBP5 inhibits GR function by slowing ligand-receptor complex translocation to the nucleus (109). It has been reported that in the HPA axis, the activation of GR inhibits the expression of CRH and ACTH, thus restraining overactivation of the HPA axis (110). Although GR activation increases the expression of *FKBP5*, the increased binding of *FKBP5* to the GR suppresses GR activity in a negative-feedback way. Thus, alterations in *FKBP5* hinders this negative feedback loop and induces “glucocorticoid resistance” (111).

Genetic variants of *FKBP5* impact gene expression. For example, the SNP rs1360780, which has been associated with a change in the three-dimensional structure of the genetic locus, influences the physical contact between RNA polymerase and the transcription start site, as well as the hormone responsive elements (HREs) located in intron 2 (112, 113). Consequently, *FKBP5* genetic variants may induce differential health stress-related outcomes *via* their influence on GR sensitivity (114), the HPA axis, and subsequent regulation of neuronal function and synaptic plasticity (115). Exposure to childhood trauma has been shown to interact with the rs1360780 risk allele (T) to increase risk for a number of psychiatric disorders (115). It has been proposed that rs1360780 risk allele carriers show an increased *FKBP5* mRNA response on exposure to stress (through enhanced binding of the promoter and the intron 2 glucocorticoid response element GRE), along with decreased negative feedback signal to the GR, which induces prolonged cortisol secretion. Enhanced cortisol secretion induces decreased DNA methylation in the intron 7 GRE, which in turn further enhances the activation of *FKBP5* (116). This dual genetic and epigenetic disinhibition may increase FKBP5 levels and induce downstream changes in cellular and circuit function to a level that promotes risk for psychiatric disorders (116). For example, in major depressive disorder (MDD) patients who have been exposed to childhood trauma, the risk allele (T) of rs1360780 has been associated with a lower methylation level of *FKBP5* in peripheral blood cells, and lower methylation of *FKBP5* has been linked to functional as well as to structural alterations in the inferior frontal orbital gyrus (117). This region of the brain belongs to the anterior limbic and paralimbic structures and plays an important role in response inhibition and cognitive function (118). Also, alterations of this area have been associated with symptoms of PTSD induced by childhood sexual abuse (119). Interestingly, it has been found that the *FKBP5* rs3800373 minor allele alters the secondary structure of *FKBP5* mRNA, decreasing the binding of a stress- and pain-associated microRNA, miR-320a. This results in relatively greater *FKBP5* translation, unchecked by miR-320a, increasing glucocorticoid resistance and increasing vulnerability to stress such as posttraumatic pain (120).

##### MAOA

Other genes that have been associated with the effects of childhood trauma are the *monoamine oxidase (MAO)* gene (located on the X chromosome), encoding the mitochondrial bound isoenzyme MAO A and B, which break down monoamine neurotransmitters such as dopamine, serotonin and noradrenaline (121). This degrading function of MAOA and MAOB is essential for the maintenance of synaptic transmission and thus proper motor control, emotional regulation, and cognitive function (122). There are more data relevant to this review on *MAOA* than on *MAOB*.

In 1993, it was reported that a point mutation in exon eight of the *MAOA* gene (leading to a premature stop codon) contributes to Brunner Syndrome, with prominent aggressive behaviors (123). Polymorphisms in *MAOA* have in fact been associated with antisocial behavior in general (27, 124), as well as with panic disorder (125), restless legs syndrome (126) and attention control (127).

The variable number tandem repeats (VNTRs) in the *MAOA* gene have been associated with differential health outcomes after stressful life events. VNTR may be variously defined, generally referring to short tandem repeats of 20–100 nucleotides. They regulate gene expression and have been associated with human diseases (128) such as spinocerebellar and Friedreich ataxia, fragile X syndrome, Huntington’s disease (128), and other psychiatric disorders.

There is a VNTR comprising CCCCTCCCCG (known as the “A repeat”) and CTCCTCCCCG (known as the “B repeat”) of a 10-base pair unit near the transcription start site (TSS) of *MAOA* that contributes to antisocial behavior after exposure to childhood trauma in females (129). The first six repeats are the same within different individuals, ABABAB; variants occur in at or after the seventh repeat. For example, seven repeats (7R) is ABABABA, and 10 repeats (10R) is ABABABAAAA. The risk allele (comprising 10 repeats) is associated with lower *MAOA* activity. Lower *MAOA* activity, which is associated with higher levels of the relevant neurotransmitters, has been associated with increased risk of conduct disorder and antisocial behavior (130, 131). Another well-studied VNTR in the *MAOA* gene is the 30 base pair (bp) upstream VNTR (u-VNTR) with a repeat sequence of 5’-ACCGGCACCGGCACCAGTACCCGCACCAGT-3’ (132). The risk allele is three repeats, which has been associated with a significantly decreased level of *MAOA* expression (133). Similarly, maltreated children with the risk *MAOA* u-VNTR genotype develop conduct disorder, antisocial personality, and violent criminality in adulthood (131).

Moreover, different genetic variants have been associated with differential methylation status of *MAOA* and corresponding phenotypes. For example, nine-repeat (9R) carriers (the lower risk allele) of the 10 bp *MAOA* VNTR show a lower methylation level in the *MAOA* promoter in females (129). In regard to the 30 bp u-VNTR, carriers of the lower-MAOA-activity variants (i.e., the higher risk alleles such as 3.5) had a higher risk of depression with histories of childhood trauma in females compared to those who without trauma histories, and the overall methylation of *MAOA* was reduced in depressed patients (130). Interestingly, the lower-activity MAOA-allele (3.5 repeats) of the *MAOA* u-VNTR has been associated with other epigenetic modifications, such as *NR3C1* hypermethylation after childhood trauma (130).

In monozygotic twin studies, hypomethylation of all *MAOA* CpG sites has been negatively associated with depressive symptoms, but not with childhood trauma; whereas, hypermethylation of the *MAOB* gene shows a nominally significant association with childhood sexual abuse (134).

##### NR3C1

Another well-studied candidate in the HPA axis is the GR gene: *nuclear receptor subfamily 3 group C member 1 (NR3C1)*. *NR3C1* encodes the GR. The binding of glucocorticoid to the GRs plays important roles in glucose homeostasis (135) and regulates the stress response through both genetic (136) and epigenetic mechanisms (137). Childhood trauma and early stress alter the methylation status of this gene and its expression.

Research has shown that altered methylation status in this gene has been associated with childhood trauma, especially the CpG sites located in the noncoding first exons of *NR3C1* (138). In a rat model, pups who received less early nurturing behaviors (low licking and grooming (LG) and arched-back nursing (ABN) from the mother rat) presented significantly higher levels of methylation in the exon 1_7_ GR promoter nerve growth factor-inducible protein A (NGFI)-A binding site (139). Since NGFI binding decreases GR expression, this alteration is thought to be associated with impaired regulation of the HPA axis (140, 141).

In humans, it has been shown that experiencing childhood trauma increases methylation of *NR3C1* (142). In Melas and colleagues’ study, one specific type of childhood trauma (parental death) was associated with hypermethylation of the *NR3C1* CpGs close to the NGFI-A binding site, at, in association with the L-allele (3.5 repeats) of the *MAOA* u-VNTR in salivary DNA samples (130). In postmortem brain tissue (hippocampus) from those who have died by suicide, there was decreased expression of GRs, along with enhanced cytosine methylation of the *NR3C1* promoter in those with a history of childhood trauma. Also, in the same group there was decreased NGF1-A transcription factor binding and NGF1-induced gene transcription (137). Labonte and colleagues also investigated methylation and *NR3C1* expression in postmortem (suicide) brains. In the hippocampus, total GR expression, and the 1_B_,1_C_, 1_H_ GR RNA variant levels decreased with history of childhood trauma. Site-specific methylation showed that the methylation of variants 1_B_ and 1_C_ was negatively associated not only with their expression but also with total GR mRNA level. Variant 1_H_ was associated with effects in the opposite direction (143). Other tissues, such as cord blood, peripheral blood, buccal epithelial cells and placental tissues were also tested, and the majority of them showed similar results in regard to enhanced methylation of *NR3C1* in association with early life adversity (138, 144).

Stressful life events occurring slightly later in life, such as in adolescence, are associated with a further independent increase in methylation of *NR3C1* (142). Interestingly, the effects of methylation within the *NR3C1* promoter can be sex-specific. Vukojevic et al. showed that enhanced methylation of *NR3C1* promoter at the NGFI-A binding site has been associated with less intrusive memory, and thus decreased risk of PTSD among survivors of the Rwandan genocide, but only in males (145).

In a recent study in mice, hemizygosity of a deletion of *nr3c1* (*nr3c1*^-/+^ heterozygote) has been associated with changes in DNA methylation in fetal placenta, and these changes have been associated with methylation changes in the adult prefrontal cortex, as well as with increased anxiety-like behaviors in the same animals (146). In addition, hydroxymethylation modifications of n*r3c1* have been suggested to be involved in the stress response pathway. Li and colleagues reported that acute stress induces accumulation of 5-hmC in the *nr3c1* 3’ untranslated region (UTR) in the mouse hippocampus (147). Further investigation of molecular mechanisms involving 5-hmC and childhood trauma in not only *NR3C1* but also in other genes could be productive.

##### HTRs and SLC6A4

Serotonin or 5-hydroxytryptamine (5-HT) is a monoamine neurotransmitter. It can be found in the gastrointestinal (GI) tract, blood platelets, and the CNS (148). In the CNS, serotonergic neurons are widely distributed in the brain, especially the limbic system (149). Serotonin contributes to brain development (149) and to maintenance of normal brain function. It promotes neural and glial cell growth, differentiation, survival and synapse formation (150). Alterations in the serotonin system have been associated with structural and functional changes in the brain (149). Stress induces brain serotonin turnover (151, 152). Excessively raised serotonin is associated with neurotoxicity (153). Stress-induced serotonin turnover has been associated with conditions such as impulsive violence (154), depressive symptoms (155), and substance dependence (156).

The *HTR* genes encode serotonin receptors, which are widely distributed in the CNSs including the prefrontal, parietal, and somatosensory cortices as well as the olfactory tubercle. Variants in these gene have been associated with differential treatment responses and with various psychiatric disorders, such as panic disorder (157), impulsive disorder (158), PTSD (159), and eating disorder (160). In children, *HTR2A* variants are related to differential risk of being hyperactive (161) with harsh parenting styles (162, 163) or after experiencing childhood abuse (164). It has been reported that early life adversity alters *HTR2A* methylation, and the effects were allele-specific. Contextual stress experienced in childhood induces enhanced *HTR2A* methylation at site-1420, in those of A/A genotype at rs6311- (-1438 A/G). Moreover, enhanced methylation of *HTR2A* at site-1420 was negatively associated with PTSD and depression; whereas, those of G/G genotype presented decreased methylation (165). Notably, hypermethylation of site-1420 has also been found in schizophrenia and bipolar patients (166). In the *serotonin 3A receptor (HTR3A)* gene, the mother’s life-time exposure to interpersonal violence is associated with altered methylation in the *HTR3A* gene, which has been associated with their children’s secure base distortion (167). In addition to the *HTR2A* and *HTR3A*, there are several other serotoninergic genes that undergo epigenetic modifications and have been associated with childhood trauma, such as that encoding the serotonin transporter.

These serotonin transporter (responsible for serotonin reuptake) encoded by *SLC6A4* (also known as the *5-HTT* gene) is in fact a frequently studied candidate in psychiatric genetics and epigenetics. Higher methylation of *SLC6A4* has been associated with childhood trauma, and with worse clinical presentation in MDD (168). In women, there is a significant association between sexual abuse and *SLC6A4* hypermethylation, which has been linked to antisocial behavior (74). In newborn babies whose mothers have depressive symptoms in the second trimester, methylation at *SLC6A4* promoter CpG sites is lower in both the newborns and in the mothers compared to controls (169). Methylation also mediates allele-specific cortisol response patterns of the *5-HTT linked polymorphic region* (*5-HTTLPR*) (rs25531) (170). The *5-HTTLPR*, consisting of a 20 to 23 bp GC-rich VNTR repeated 14 times in the short allele (S) and 16 times in the long allele (L), is located 1 kb upstream of the *SLC6A4* gene. The short variant is associated with reduced *5-HTT* expression (171). The S/S *HTTLPR* genotype has been associated with increased risk of depression and suicide attempts after stressful events (172), as well as with adult depression after childhood trauma (173). In Alexander and colleagues’ study, it was showed that those of S/S genotype with lower methylation level exhibited higher cortisol response; while the association of the *5-HTTLPR* short allele with enhanced cortisol response disappeared at a higher *SLC6A4* methylation level (170).

##### BNDF

BDNF has been investigated not only for association with childhood trauma, but also for association with mental health outcomes such as psychotic or depressive symptoms (174–176). *BDNF* encodes a neurotrophin, which promotes the growth, differentiation and survival of neurons. BDNF is also involved in neuroplasticity. Structural brain changes are seen after trauma and BDNF is hypothesized to be involved in these (177). For example, early isolation (one type of ACEs) causes decreased BDNF levels in the amygdala and infralimbic cortex; however, the combination of resocializing and the antidepressant fluoxetine reverses the reduction of *bdnf* in rodents (178). In a rat model, early stress (being abused by caretakers) induces enhanced *BDNF* methylation and decreased *bdnf* gene expression in the prefrontal cortex in exon 9 and 14, which includes the transcription start site (TSS) and cyclic adenosine monophosphate (cAMP) response element and the enhanced methylation persists until adulthood (179). In rodents, the *bdnf* gene contains 9 noncoding exons, each of which can be linked to the protein coding exon IX (9) after splicing, and transcription can be initiated before the protein coding exon in the intronic area. Exon-specific transcription is tissue-specific. Importantly, methylation-induced altered gene expression of BDNF is also cell-type specific (180, 181).

In humans, childhood trauma has been associated with decreased serum levels of BDNF (182). Also, childhood maltreatment induces alterations in *BDNF* promoter methylation (75). It has been shown that a lower maternal care condition is associated with higher *BDNF* DNA methylation levels (183). Furthermore, differential *BDNF* methylation has been associated with structural brain changes. For example, socioeconomic disadvantage has been negatively associated with *BDNF* DNA methylation, specifically at the exon IV promoter site, and this lower level of *BDNF* methylation has been linked to greater thickness of the lateral orbitofrontal cortex (IOFC), medial frontal cortex and decreased thickness of the bilateral IOFC in adolescence (age 12–13) (184). These brain areas are relevant to decision-making, emotion, and memory processing.

In addition, BDNF works synergistically with other genes after childhood trauma, such as the 5-HTTLPR (182), noradrenaline/norepinephrine transporter (NET) and corticotropin releasing hormone receptor 1 (CRHR1) genes (185), as well as tryptophan hydroxylase (TPH) 2 (186). In fact, the BDNF receptor TrkB and GRs, as well as mineralocorticoid receptors, are coexpressed in hippocampal neurons. Additionally, as mentioned above, BDNF directly regulates the HPA axis. The administration of BDNF in vivo induces increased CRH level and reduction of BDNF or its receptor normalizes the CRH level and thus, the HPA axis. This cross-talk between BDNF and CRH may be at least partly mediated by the CREB and MAPK pathway and is involved in the enhancement of fear memory under stress (187).

##### Other Candidate Genes

There are other candidate genes with at least some data in childhood trauma and epigenetic alterations, such as *COMT*, *IL-6* (188), and *OXTR* (189).

The catechol*-O-*methyltransferaseenzyme encoded by the *COMT* gene on chromosome 22q11.2 (190), is involved in the metabolism of catecholamines including the neurotransmitters dopamine, adrenaline, and noradrenaline, in reactions that involve the transfer of a methyl group from *S*-adenosyl-methionine (SAM) to a hydroxyl group (191). There appear to be epistatic effects between *COMT* and *NR3C1* on working memory (192). In addition, methylation of the *COMT* promoter has been associated with a change in prefrontal cortical connectivity in schizophrenia (193), as well as in depression (194).

*Interleukin 6 (IL-6)* encodes the IL-6 protein, which is a proinflammatory cytokine. Alterations in IL-6 have been associated with psychiatric disorders, such as depression (195). In addition, patients with schizophrenia and a history of childhood trauma have a pro-inflammatory phenotype (196). Inflammatory factors can in fact be regarded as mediators that connect the environmental stimulus of childhood trauma with clinical symptoms. Changes in the *IL-6* methylation has been associated with childhood trauma related phenotypes. In African American men, there was an association with decreased methylation of *IL-6* and enhanced IL-6 protein level after childhood trauma (197). Importantly, altered expression of *IL-6* can be associated with other genetic variants that are involved in neural pathways. For example, women who carry two short alleles of the *5-HTTLPR* present a higher IL-6/IL-10 ratio when dealing with stress (198).

Oxytocin is a neuropeptide hormone facilitating labor and breastfeeding in mammals. In the brain, oxytocin receptors (OXTRs) are expressed mainly in the central nucleus of the amygdala (cAmyg) and the ventromedial nucleus of the hypothalamus (VMH) (199). The cAmyg regulates the fear response (200) while the VMH regulates a range of behaviors including female sex behaviors (201). Oxytocin and its receptor are involved in the regulation of attachment, social behavior and the stress response (202). In a recent study, there was hypermethylation at *OXTR* CpG sites in children who had experienced childhood trauma, and hypermethylation has been associated with decreased grey matter volumes in the orbitofrontal cortex (OFC), which may be related to insecure attachment styles (189).

#### Complicated Interactions/Cross-Talk

Research has shown that altered methylation has been associated with childhood trauma-induced phenotypes. Several candidate genes (*FKBP5, MAOA, NR3C1, HTR and SLC6A4, BDNF*) have been discussed in this review. However, the actual regulatory network and mechanisms are more complicated.

Firstly, multiple functional pathways or circuits are involved in processes relevant to stress, including both the reward and the fear circuits, emotional regulation and executive cognitive function. Secondly, in the HPA axis, molecules and their receptors interact and cross talk with each other. Thirdly, there are potential gene by gene, gene by environment, gene by epigenetic modification, and even epigenetic by epigenetic modifications interactions. All these components influence stress-related phenotypes.

For example, the reward pathway/circuit in the brain has been associated with trait optimism, which has been associated with resilience after stress (203).There are two main reward pathways in the brain: the mesocortical dopamine pathway and the mesolimibic dopamine pathway. Glutamatergic and GABAergic connections are also involved in the reward circuit (204). Similarly, glutamatergic and noradrenergic neuronal signalling (203) and dopaminergic connections participate in neuronal regulation in the fear circuit. In addition, adrenergic receptors (205) and GRs (206) are also involved in fear conditioning. The serotonergic and noradrenergic systems have an established role in mood regulation, while the former is involved in motivation as well, with both anxiogenic and anxiolytic effects (207). Dopamine is relevant to mood regulation too. Enzymes regulating these pathways, such as COMT, MAOA and MAOB, regulate these phenotypes.

At the molecular level, there are different levels of cross-talk. For example, the dopamine D_1_ receptor interacts with glutamate-mediated excitatory neurotransmission through protein-protein interactions (208). In addition, serotonin signalling, has been reported to interact with cannabinoid receptors (209). Acting as retrograde synaptic messengers (210), the endogenous cannabinoids, such as anandamide, sleep-inducing substance oleamide (211) and palmitoylethanolamide (212), regulate numerous biological processes such as neuronal migration (213), learning, memory (214), pain processing (215), motility (216), and emotional-and reward-related processing (217–219).Further, both serotonin and endocannabinoids are involved in stress-related phenotypes, such as anxiety (212). In addition, serotonin is also involved in the regulation of GRs, such as in primary hippocampal cell cultures (220) and in the rat brain (221). At the genetic level, it has been reported that different genotypes of the *5-HTT* gene has been associated with the altered GRs’ mRNA level under conditions of childhood adversity (222). A variant in *MAOA* gene is associated with differential *NR3C1* methylation (130). For *BDNF*, its expression level responds to stress-related HPA axis activation. Moreover, there is a feedback loop whereby directly regulates CRH, and thus, the HPA axis (187). Besides, as mentioned above, multiple other genes, act in concert with *BDNF* (185). These genes further interact with other genetic/epigenetic variants to form a sophisticated molecular and functional network, which has not yet been fully characterized. For example, *TPH2* also interacts with the *adenosine deaminase, RNA specific B1* (*ADARB1*) gene, which affects pre-mRNA splicing. The interaction of these two genes predicts risk of suicide attempts after childhood trauma (223). A given neurotransmitter/neuronal pathway may conduct more than one function, e.g., glutamate signaling has been associated with both activation and inhibition of the HPA axis through inotropic and kainite/group I metabotropic receptors respectively (224). Interestingly, cognitive therapy and cognitive reappraisal decreases amygdala and HPA activation in response to stress (225), suggesting that there is some “flexibility” in stress-related psychiatric phenotypic presentations. Hence, molecular mechanisms of the HPA axis and the stress response pathway more widely are not only highly complex and orchestrated but also require further illumination.

## Limitations and New Directions

### Limitations

Limitations exist in this field. Even though numerous studies have been done, evidence of associations between epigenetic/epigenomic alterations and differential health outcomes induced by childhood trauma are limited (226). Additionally, there are inconsistencies in the field. For example, the association between childhood trauma and *NR3C1* methylation has not been consistently replicated (138) and likewise the differences in *SLC6A4* methylation between trauma- and nontrauma-impacted groups (104).

The full complements of molecular mechanisms involved in childhood trauma related health outcomes remain to be elucidated (31). As mentioned above, a further complication is the possibility of coordinate regulation of epigenetic processes in more than one gene/pathway. In addition, there may be pleiotropic or polygenic effects. Pleiotropy means that a gene is associated with more than one phenotype (e.g., the association between *disrupted in schizophrenia 1 (DISC)1* mutations and various psychiatric disorders) (227), and polygenic means that one phenotype may be influenced by several genes (e.g., AOB blood type systems). Moreover, metastable epialleles, differential expression of alleles induced by epigenetic modification during early embryonic development have been identified in genetically identical individuals, and these may also induce phenotypic changes (228). Additionally, study heterogeneities may have limited the conclusions possible in this data synthesis.

#### Phenotypic Heterogeneity

Between study heterogeneity includes the investigation of different types of childhood trauma. Research has shown that different types of trauma stimulate different brain areas (77). Although psychological trauma might induce similar biological responses to physical trauma (229), the affected brain areas are different: physical stressors mainly impact the brainstem and hypothalamus (230); whereas, psychological stressors mainly impact regions that regulate emotion, learning, memory and decision making, e.g., the hippocampus, the amygdala and the prefrontal cortex (231, 232). Moreover, long-term stress and acute stress have different effects on the brain. Trauma timing, and frequency also impacts differential health outcomes owing to neurodevelopmental stages (233). However, the exact timing as well as the frequency are difficult to reliably record, since the most common type assessment for childhood maltreatment is retrospective self-report, which may map relatively poorly on to prospective assessments (234).

In addition, phenotypic measurement and diagnosis for children who experience childhood trauma may be ambiguous. In diagnosis, children exposed to childhood trauma may develop PTSD, but the potential outcomes are not limited to PTSD (235). Even with PTSD, there are arguments about the diagnostic criteria in DSM-5 (e.g., lack of connection between exposure to stressor and some specific symptoms, some very-well-documented symptoms failing to be captured in DSM-5, and lack of extensive field trial data (236)). Consequently, the term ‘complex PTSD’ has been put forward to describe complicated traumatic outcomes not captured by standard PTSD (237). Importantly, in behavioral measurement, it is necessary to develop appropriate mathematical models and measurements to correctly quantify within- and between-individual variability (238).In behavioral studies, it is hard to define associations between single genetic/epigenetic variants, as behavioral traits are usually controlled by multiple genes (239). In the definition of childhood trauma induced phenotypes, cultural and ethnic differences may bring additional between study heterogeneity (240). There are other factors such as sex/gender differences in response to stress (241, 242), and the use of different tissues (saliva, cord blood, whole blood and peripheral blood) by different researchers (243). The latter brings complexity to data comparisons since the epigenetic signature differs between and within tissues (244).

Crucially, more than one trait contributes to health outcomes after experiencing trauma. The same genetic/epigenetic modification may impact differently on different traits in one individual. For example, 7 repeat (7R) carriers of the *DRD4* exon III VNTR exhibit the highest sensitivity toward parental-induced stress (245); however, they also show a higher level of emotional resilience due to the association between the 7R and specific personality types (246).

#### Methodological Heterogeity

Although epigenetics is not a novel concept [the first scientific hypothesis of epigenetics was put forward by Malpighi (247) in 1673, with a key milestone of epigenetic development in 1975 by Riggis (248), Holliday and Pugh (249)], and may simply mean inherited altered gene expression states, it may also refer to inter- versus trans-generational effects, where the former refers to transmission across one generation, and the latter to transmission across multiple generations (250). Historically, these terms have been ambiguously defined (247, 251, 252). This has led to misunderstandings as well as to bias in methodologies and interpretations, especially in interdisciplinary research (253). Indeed, inherited epigenetic patterns (254–256) and environmental factors (257, 258) other than childhood trauma (such as heavy metals (259), parenting style, and early trauma such as maternal separation (260)) may all impact the epigenetic pattern and hence childhood trauma-induced differential health outcomes. However, how much these changes are due to these factors, and to what extent, remains, unclear (261).

In regard to methylation, except for CpG methylation, there are some non-CpG methylations, such as CpA, CpT, and CpC. These are expressed in cell types such as pluripotent stem cells, oocytes, neurons, and glial cells. Importantly, these non-CpG methylations are critical in maintaining neuronal function and are thus involved in neurological disorders (262). Kigar and colleagues posited that adenosine methylation could be regarded as an epigenetic marker of mammalian early life stress (263). However, more research is needed in regard to the above non-CpG methylations, and also that of 5-hmC. As for non-coding RNA, and histone acetylation, there are to date few investigations of associations between these and childhood trauma. Furthermore, the various epigenetic mechanisms, such as methylation, histone modification and noncoding RNA, while often studied one by one, may cooccur and act in concert.

Research has shown that the effects of trauma can be intergenerationally passed on through epigenetic mechanisms, such as methylation (264). Specifically, childhood trauma has been associated with alteration in methylation patterns in human sperm, which may induce intergenerational effects. Further such analyses in larger samples are required (265). Importantly, in addition to epigenetic modifications, other factors, such as epimutations (an mutation occur at the epigenetic level), fetal reprogramming (266), and even gut microbiome transfer (267) may induce intergenerational phenotypic changes. It is challenging and costly to investigate/exclude all of these factors in one human study.

### Sex/Gender Differences

Sex/gender differences exist in this research field. In stress-related psychiatric disorders, there are sex-associated differences in incidence, symptoms and treatment response (268). For example, in PTSD, the life time prevalence in females (10-12%) is 2-3 times higher than that in males (5-6%) (269). Similarly, depression is more common in females than males (268). Interestingly, both sex- and gender-related concepts contribute to these differences (270).

There are multiple reasons that may explain these phenomena, such as differential traumatic exposures, cognitive factors, coping strategies and biological factors between different sexes. There are also fundamental sex-dependent brain differences between males and females, e.g., the size of vasopressin (AVP) neurons (271). Moreover, when dealing with stress, males and females present different sex-specific cortico-striatal and limbic patterns. In the work of Cahill and colleagues (272), men showed greater activation of the right amygdala; whereas, women showed greater activation of the left amygdala when facing stress (272). In addition, brain connectivity in response to stress also differs by sex: e.g., there was greater connectivity between the anterior and dorsal anterior insula, as well as between the anterior and dorsal anterior mid-cingulate in males than females after stress (273, 274).Similarly, Helpman and colleagues showed that males present overactivation and increased connectivity of salience hubs (including anterior insula (AI) and dorsal anterior cingulate cortex (dACC)); whereas, females show an overactive and possibly enlarged amygdala. In addition, males lose more grey matter after stress in limbic system structures (prefrontal cortex, amygdala and the hippocampus (275). These differences contribute to differential fear processing, emotional regulation and decision-making. Moreover, males and females cope with stress differently. For example, when facing traumatic stress, females tend to be more emotion-focused and to use more palliative coping skills than males. Also, females tend to seek social support more and benefit more from psychotherapies (269). Differential stress-related phenotypes between males and females are also related to the gonadal hormones, which play important roles in the establishment, activation and regulation of the HPA axis (276). In animal models, both female rats and mice exhibit more robust responses of the HPA axis (such as a higher level of ACTH), owing to circulating estradiol. In rats, progesterone and estrogen have been shown to directly impact the stress response in females (277). Epigenetic modifications are also involved in gonadal hormone setting up and maintenance of sex differences in the brain, even before puberty (278). In rodents, it has been shown that females have significantly higher level of methylation in the estrogen receptor-alpha (ER-α) promoter than estradiol treated females or males (279). Note that, early exposure to estradiol induces masculinization/defeminisation (280, 281). Interestingly, these sex-dependent epigenetic changes are dynamic across the lifespan (279).

Current studies in regard to epigenetics and sex-dependent phenotypes mainly focus on steroid hormones and targets related to the HPA axis, such as *NR3C1*, and majority of them are association studies, e.g.,the enhanced methylation of *NR3C1*and PTSD risk (145). There are also neurotransmitter specific effects in sex differences. For example, in a study by Oswald and colleagues, the availability of the dopamine D_2_ receptor (D2R) has been associated with childhood trauma and pleasant drug (amphetamine) effects. In males, there was a positive association between childhood trauma and pleasant drug effects but not in females (282), which suggests that there may be by sex differences in the reward pathway after childhood trauma (283). Autonomic systems are also different between males and females (284), which may also contribute to sex differences in stress-induced phenotypes. Groleau and colleagues reported higher methylation of the *DRD2* promoter in women with an eating disorder and a history of childhood trauma versus those without such a history (285). Comparison studies between both males and females are limited, probably owing to the different prevalence within different sexes; in some studies with both females and males, the sample sizes were too small to have enough power; the comparison study between the differences of self-identified gender and biological sex, which may provide us the biological and psychological effects about sex-dependent stress responses, are limited; in addition, current studies are mainly focused on the candidate genes that are related to steroid hormones, and they are mainly association studies, which can’t provide the information about the causality. Research about more in-depth molecular mechanisms between different sexes, and their interactions with other genetic, epigenetic, as well as environmental factors is limited. Thus, the epigenetic contribution to sex-dependent stress-related phenotypes is still filed for research exploration.

By sex and gender differences are still relatively new areas of research, and hence replications are required and interactions between the above components remain to be explored (285–288).

#### Technical Limitations

Interestingly, it has been reported that epigenetic patterns and phenotypic changes can be induced by a single genetic variant, combined with random epimutation (289). Hence, it has been recommended that when investigating epimutations and phenotypic changes, the DNA sequence, replication, GC%, and the topological structure of chromosomal bands, especially in unstable genomic areas, should be first analyzed (290) - in an integrated combined “omic” approach. Chromosomal banding was first used with light microscopy and divides chromosomes into regions visible at that level of magnification. These regions include G bands, which have a lower number of genes and lower gene expression level, which replicate late in the cell cycle, and R bands, which have a higher gene number, GC content and expression levels (291). Alterations in the topological structure of chromosomal bands have been associated with changes in gene expression and thus with phenotypes (292–294).

In epigenome-wide association studies (EWAS), although these provide the opportunity to investigate epigenetic variants (methylation, noncoding RNA and histone modification) on a genome-wide level, which could assist with identification of disorder-related markers in different populations (295), the individual CpG sites detected by array methods are limited (296). Genome-wide sequencing approaches can be helpful, but DNA methylation sequencing at a depth to reliably detect the small changes often observed in mixed tissues in human studies is very costly. Targeted assays with high sensitivity covering functionally relevant regions could be an interesting complement here (297). Nonetheless, issues such as cost, speed of delivery, errors of variant annotation, logical and methodological issues (e.g., the appropriate selection of the cohort, population stratification and statistical approaches) remain in human genomic and epigenomic studies (298, 299). Consequently, multiple validations *via* more than one method might bring more reliability.

### New Directions

New technologies and strategies have emerged in this field. For example, the nanopore sequencing framework, able to distinguish five types of methylation variants with high-throughput (300). The usage of this technology reduces sample preparation processes and increases the detection speed (300). In addition, nanopore sequencing is able to detect 5-hmC (301), which is not adequately covered by traditional array/bisulfite sequencing methods. We suggest a more in-depth investigation of molecular mechanisms including 5-hmC in relation to childhood trauma related effects.

In living cells, fluorescence recovery after photobleaching (FRAP) has been reported to be able to detect histone mobility (302), which permits real-time investigation of dynamic histone modification. In regard to chromatin structure, Stevens and colleagues reported that the combination of chromosome conformation capture (3C) and tagged fluorescent imaging was able to detect the folding of a genomic sequence <100bp in a single cell (303). This provides the opportunity to investigate how epigenetic modifications dynamically and spatially mold chromosomes and thus, cellular function and related phenotypes in animal models *in vivo*.

In addition, the CRISPR-CAS9 system can be used to study targeted genetic/epigenetic variant-induced phenotypic changes in animal models. In fact, usage of a modified CRISPR-cas9 system has been expanded beyond genome editing, to RNA targeting, chromatin topology, chromatin imaging, and developmental trajectories as well as to lineage tracing (188, 304). Since the effects of childhood trauma are neurodevelopmental stage-sensitive, a tracing-based technique may provide us with information about when sensitive periods toward different stress are, and how stress impacts on neuronal differentiation (305). The CRISPR-cas9 system can also be used as an effective tool to edit the epigenome (306). Liao and colleagues reported that the endogenous gene was activated *via* trans-epigenetic remodelling by using a CRISPR-cas9 system, and phenotypic changes were observed in acute kidney injury, type 1 diabetes and Duchenne muscular dystrophy rodent models (307). Thus, epigenome editing may help us to better understand the molecular mechanisms in diverse stress-related phenotypes with known targeted sequences. More in-depth molecular insight may also be helpful for improving the definitions and diagnoses of different psychiatric phenotypes.

Given the cell-type specificity of epigenetic changes, achieving single cell-, or at least single cell type-resolution is also an important goal. Single cell sequencing is able distinguish methylated changes in different cell types, and thereby reduce in errors/bias. Using such techniques in combination with sex-dependent stratification, different network mechanisms in males and females may be distinguished. So far, a number of single cell sequencing techniques have in fact been developed to facilitate investigation of methylation (308). For example, single-cell nucleosome, methylation and transcription sequencing (scNMT sequencing), combining epigenome and transcriptome data, are able to detect several “layers” of epigenomic and molecular dynamic coupling processes (309). Psychiatric disorders are more regarded as network dysfunctions (310). As mentioned above, focusing on only one cell type, brain area or neuronal pathway may not be sufficient. Thus, a combination of single cell sequencing and a pathway approach to the analysis of methylation patterns similar to network analysis in genomics (as exemplified by weighted gene coexpression network analysis or WGCNA) could be fruitful in this field.

Furthermore, the assay for transposase-accessible chromatin by sequencing (ATAC-seq) is able to get access to DNA sequences in open chromatin and to produce high quality data with a low background in a high-through output way (311). When being used at the single cell level, ATAC-seq detects DNA regulatory variations, e.g., *trans*-factors, *cis*-elements, which have been associated with induction or suppression of cell-to-cell variability. Such DNA variation data can be combined with chromatin accessibility and thus form a three-dimensional informative “regulome” in the genome (312). The concept of “connectomics” put forward by Fornito and colleagues, may also benefit this field of research (313). “Connectomics” was originally characterized as brain-network topological regulation of neural activities after injury (313). The combination of the different “omic,” such as genomic, epigenomic, transcriptomic, and even connectomics studies, may form interesting perspectives about how genetic/epigenetic and their molecular and topological mechanisms impact different cells and brain areas, and thus, stress-related phenotypes. So far, combined “omic” studies such as the combination of GWAS data with enhancer enrichment profiles, RNA sequencing data (RNA-seq) and chromatin status have been utilized (314). The integration of *in vitro* cell culture and multi “omic” analysis in the investigation of human germline epigenome reprogramming has been reported, producing some hints about the origin of neuropsychiatric disorders and transgenerational inheritance (315, 316).

In summary, by using new technologies, “omic” analysis and “big data”-integration of data from different platforms in a system biology approach-bias will be reduced and understanding of molecular mechanisms will be deepened (317). In the future, integration of genomics, epigenomics, transcriptomics, proteomics, metabolomics, regulomics, and connectomics could shed light on both basic biological processes in response to childhood trauma and disorder-related mechanisms, and thereby produce innovations in mental health and addiction health service provision.

## Author Contributions

SJ conducted the literature review and drafted the paper for a course (MDGEN605). LP, EB and AC reviewed the manuscript and provided some text and suggested edits. KA reviewed the manuscript, discussed with SJ, provided some text and suggested edits.

## Funding

An Alberta Innovates Health Solutions Team Grant (Collaborative Research & Innovation Opportunities (CRIO) Population Resiliency Grant Prediction and Understanding of Resilience in Albertan Families: Longitudinal Study of Disaster Responses (PURLS)) contributed to the training of the first author.

## Conflict of Interest

The authors declare that the research was conducted in the absence of any commercial or financial relationships that could be construed as a potential conflict of interest.
